# MARCKS-dependent mucin clearance and lipid metabolism in ependymal cells are required for maintenance of forebrain homeostasis during aging

**DOI:** 10.1111/acel.12354

**Published:** 2015-05-25

**Authors:** Nagendran Muthusamy, Laura J Sommerville, Adam J Moeser, Deborah J Stumpo, Philip Sannes, Kenneth Adler, Perry J Blackshear, Jill M Weimer, H Troy Ghashghaei

**Affiliations:** 1 Department of Molecular Biomedical Sciences, College of Veterinary Medicine, North Carolina State UniversityRaleigh, NC, 27607, USA; 2 Department of Population Health and Pathobiology, College of Veterinary Medicine, North Carolina State UniversityRaleigh, NC, 27607, USA; 3 Center for Comparative Medicine and Translational Research, College of Veterinary Medicine, North Carolina State UniversityRaleigh, NC, 27607, USA; 4 Laboratory of Signal Transduction, National Institute of Environmental Health SciencesDurham, NC, 27709, USA; 5 Sanford Research, Children’s Health Research and Department of Pediatric, University of South Dakota Sanford School of MedicineSioux Falls, SD, 57104, USA; 6 Program in Genetics, North Carolina State UniversityRaleigh, NC, 27607, USA

**Keywords:** aging, barrier function, Clca3, cerebral cortex, ependymal cells, lipid droplets, mucin, oxidative stress

## Abstract

Ependymal cells (ECs) form a barrier responsible for selective movement of fluids and molecules between the cerebrospinal fluid and the central nervous system. Here, we demonstrate that metabolic and barrier functions in ECs decline significantly during aging in mice. The longevity of these functions in part requires the expression of the myristoylated alanine-rich protein kinase C substrate (MARCKS). Both the expression levels and subcellular localization of MARCKS in ECs are markedly transformed during aging. Conditional deletion of MARCKS in ECs induces intracellular accumulation of mucins, elevated oxidative stress, and lipid droplet buildup. These alterations are concomitant with precocious disruption of ependymal barrier function, which results in the elevation of reactive astrocytes, microglia, and macrophages in the interstitial brain tissue of young mutant mice. Interestingly, similar alterations are observed during normal aging in ECs and the forebrain interstitium. Our findings constitute a conceptually new paradigm in the potential role of ECs in the initiation of various conditions and diseases in the aging brain.

## Introduction

Ependymal cells (ECs) form the epithelial lining of the ventricular surfaces in the brain. They are highly polarized with adherens junctions at their apical interface allowing them to form a highly selective barrier between the interstitial tissue and cerebrospinal fluid (CSF) in the ventricles (Del Bigio, 2010; Johanson *et al*., 2011). Compromise in ependymal function has been correlated with pathogenesis of ventriculomegaly, neurocognitive deficits, and neurodegenerative diseases such as schizophrenia, attention deficit disorder, and Alzheimer’s disease (Silverberg *et al*., 2003; Palha *et al*., 2012). Despite the potential significance of ECs, remarkably little is known regarding their biological roles and intracellular mechanisms regulating their functions.

ECs differentiate during perinatal development (Spassky *et al*., 2005; Jacquet *et al*., 2009) and lose their inherent ability to perform CSF–interstitial barrier functions during aging. The plasticity and barrier capacity of ECs likely diminishes with increased age based on several observations. For example, the ependymal layer significantly thins as astrocytes simultaneously infiltrate the layer and form tight junctions with resident ECs (Luo *et al*., 2006). Moreover, there is noticeable reduction in the density of motile cilia on the apical surface of ECs in the aged brain (Luo *et al*., 2008; Capilla-Gonzalez *et al*., 2014). These motile cilia are necessary to direct the flow of CSF along the ventricular surface and generation of molecular gradients of factors that circulate in the CSF and are filtered into the brain interstitium (Sawamoto *et al*., 2006). In addition, aged ECs accumulate lipid droplets in mice (Bouab *et al*., 2011; Capilla-Gonzalez *et al*., 2014), indicative of either loss of metabolic efficiency or increase in lipid uptake from the CSF and interstitial brain tissue. However, whether loss of ependymal integrity during aging can induce interstitial defects has yet to be tested. Testing this hypothesis requires new models in which ependymal cell functions can be modified to mimic aged ependyma, that is, ones in which ECs are precociously aged.

In this study, we found that the myristoylated alanine-rich protein kinase C substrate (MARCKS) is critical for previously unappreciated biological functions in ECs related to their aging. Selective deletion of MARCKS in ependyma results in precocious emergence of biomarkers for aging, both in ECs and in the brain interstitium. MARCKS is a known regulator of cell polarity, cytoskeletal signaling, cell migration, and embryonic brain development (Stumpo *et al*., 1995; Weimer *et al*., 2009), and plays a role in vesicular trafficking in lung epithelial cells (Park *et al*., 2007). Functions of MARCKS are tightly regulated through phosphorylation by conventional, novel, and atypical isoforms of protein kinase C (PKC) (Hartwig *et al*., 1992; Herget *et al*., 1995). Dephosphorylated MARCKS associates with the plasma membrane, and PKC-dependent phosphorylation of MARCKS dissociates it from the plasma membrane, resulting in translocation to the cytosol (Blackshear, 1993; Swierczynski & Blackshear, 1996). Dynamic phosphorylation and dephosphorylation of MARCKS allow it to associate with a number of intracellular signaling proteins, including membrane phospholipids (Blackshear, 1993).

Although the association of MARCKS with key regulators of cell permeability and secretion has revealed a putative function in the lung epithelium (Park *et al*., 2007; Jin *et al*., 2012), its precise roles in distinct cell types of the brain remain largely unknown. Here, we demonstrate that cellular alterations in young MARCKS-null ECs correlate with age-associated elevation of lipid droplets and mucin accumulation in wild-type ependyma. These alterations reveal the significance of MARCKS for the maintenance of ependymal barrier integrity and potential secretory and/or filtration functions which indirectly impact forebrain homeostasis.

## Results

### Polarized expression of MARCKS in ECs is altered with age

**Fig 1:**
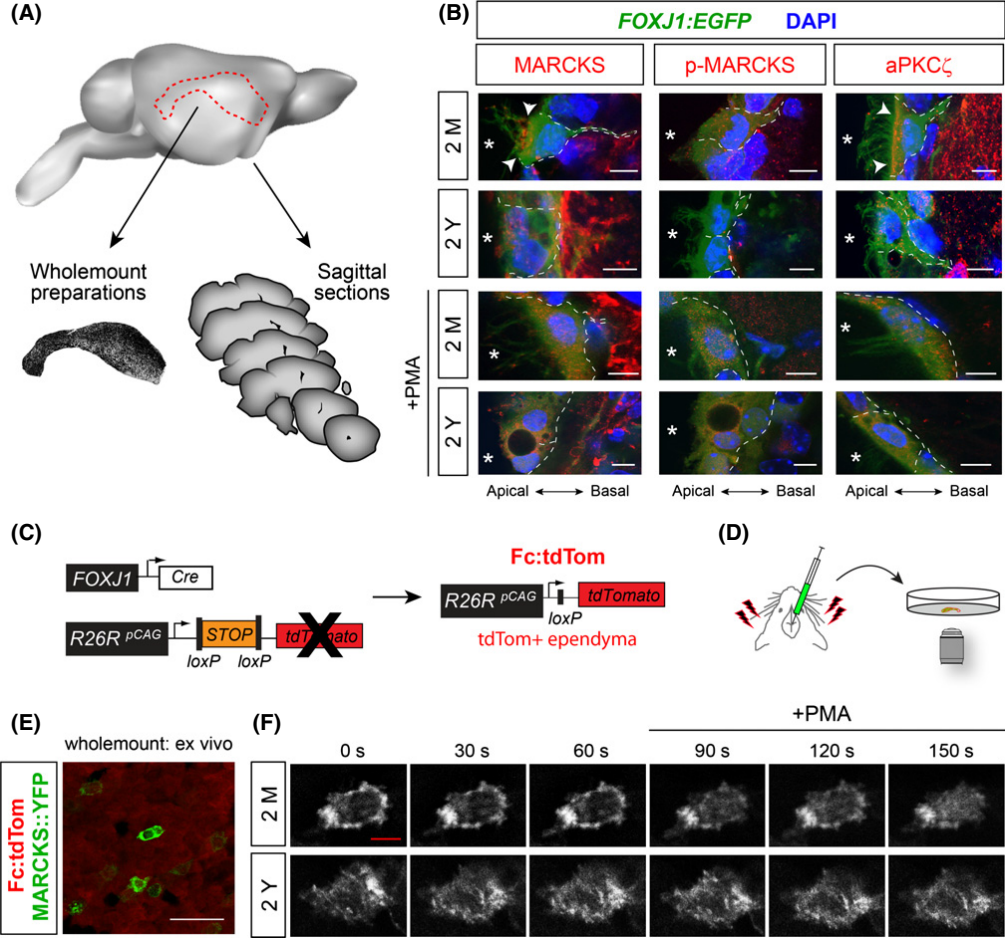
MARCKS is expressed in ECs and is internalized upon phosphorylation. (A) Approach utilized throughout the study in using cross sections and wholemounts from mouse brains for various analyses. (B) FOXJ1:EGFP transgenic mice with EGFP labeled ECs (green, outlined with white dotted lines) were utilized for immunofluorescence analysis of MARCKS (red), phosphorylated MARCKS (p-MARCKS, red) and atypical PKC zeta (aPKC_ζ_, red) in young (2M) and old (2Y) brains. Nuclei are labeled with DAPI (blue); asterisks indicate the lumen of the ventricles. Note that immunoreactivity outside ECs is highly variable, which results in differences seen in these images and do not necessarily reflect effects of aging. ‘+PMA’ marks sections obtained from 2M and 2Y FOXJ1:EGFP brains which were intraventricularly injected with PMA and perfused 5 min later. MARCKS, p-MARCKS, and aPKC_ζ_ localizations were significantly altered upon PMA stimulation. Scale bars: 10 μm. (C) Diagram of approach to selectively label ECs *in vivo* with a FOXJ1-cre-dependent tdTomato reporter system (Fc:tdTom). (D) Adult ECs were electroporated with a MARCKS::YFP construct to analyze its dynamics in wholemounts *ex vivo*. (E) Low-magnification images illustrating mosaic of Fc:tdTom ECs (red) successfully electroporated with the MARCKS::YFP expression plasmid (green) in cultured wholemounts. Scale bar: 50 μm. (F) Time-lapse panels illustrate PMA-induced dissociation and internalization of MARCKS::YFP in ECs. Scale bar: 5 μm.

To investigate the function of MARCKS in ECs, we conducted *in vivo* studies using cross sections or wholemount preparations of the ependymal zone (Fig.1A). Subcellular localization of MARCKS was examined using mice in which ECs express the enhanced green fluorescent protein [*FOXJ1:EGFP*; (Jacquet *et al*., 2009, 2011)]. Antibody staining in cross sections of 2-month-old brains (2M) showed MARCKS enriched in the proximal aspects of apical cilia in ECs (Figs1B, S1; Movie S1). As phosphorylation status of MARCKS is an important regulator of its subcellular localization in various cell types, we examined p-MARCKS distribution in 2M ECs *in vivo* (Fig.1B; Movie S2). p-MARCKS, which represents only a fraction of the total MARCKS pool, is distributed throughout the cytosol away from the apical surface of young ECs.

MARCKS is a prominent substrate for conventional and atypical isoforms of protein kinase C (PKC) (Hartwig *et al*., 1992; Herget *et al*., 1995). Based on the known expression and function of atypical PKC (aPKC) isoforms in various epithelial cells (Ishiuchi & Takeichi, 2011), we reasoned that some isoforms may be uniquely expressed in ECs and utilize MARCKS as a substrate. Expression of zeta isoform of aPKC (aPKC_ζ_) was confirmed using antibody labeling, which additionally revealed that its subcellular localization mirrored the distribution of MARCKS in 2M ECs, enriched at the apical surface (Fig.1B, Movie S3).

Detailed imaging of ECs in 2-year-old (2Y) mouse brains revealed MARCKS loses its apical clustering and becomes less polarized in the cytosol, whereas p-MARCKS is sparsely distributed (Fig.1B; Movies S4 and S5). Similarly, aPKC_ζ_ loses its preferential apical localization in aged ECs and its expression level declines (Fig.1B; Movie S6). Thus, MARCKS and aPKC_ζ_ are highly polarized toward the apical surface of ECs in the young brain, a pattern that is lost in aged ECs.

### Phosphorylation status of MARCKS regulates its subcellular localization

Based on the apparent impact of aging on the polarized distribution of MARCKS and aPKC_ζ_ in ECs, we focused on determining how the phosphorylation state of MARCKS may influence its localization and function. In lung epithelial cells, phosphorylation of MARCKS is known to regulate its association with filamentous actin (F-actin) near the plasma membrane. MARCKS dissociates from actin networks and the plasma membrane upon phosphorylation by PKC (Park *et al*., 2007). To determine whether MARCKS functions similarly in ECs, 2M and 2Y FOXJ1:EGFP mice were intraventricularly injected with a pharmacological PKC activator (phorbol-12 myristate-13 acetate, PMA) and sacrificed five minutes later (+PMA; Fig.1B). Confocal analysis of cross sections revealed that MARCKS and aPKC_ζ_ become internalized in PMA-exposed ependyma, concomitant with the elevation of p-MARCKS levels in ECs as expected (Fig.1B; see +PMA in Movies S1–3). Interestingly, alterations in subcellular localization of MARCKS, p-MARCKS, and aPKC_ζ_ are less visible in 2Y ependyma in response to PMA exposure as seen in 2M brains (Fig.1B; Movies S4–6). Taken together, these *in vivo* findings indicate that MARCKS has a polarized distribution in young ECs and that phosphorylation presumably by aPKC_ζ_ may favor its internalization. The capacity for MARCKS’s subcellular mobility *in vivo* may be attenuated during aging.

To directly monitor the temporal dynamics in MARCKS’s localization following PMA-induced phosphorylation, we time-lapse imaged ECs either cultured or in wholemount preparations (Mirzadeh *et al*., 2010). To accomplish this, we utilized mice in which ECs selectively express the fluorescent reporter tdTomato (Fig.1C; *FOXJ1:Cre*, *Rosa* ^*pCAG-tdTom*^; referred to as Fc:tdTom hereafter; Jacquet *et al*., 2011). Newborn Fc:tdTom mice were intraventricularly electroporated with a construct encoding MARCKS fused to yellow fluorescent protein (MARCKS::YFP). Electroporated brains were harvested 24 hours later, and ependymal cultures were established. Time-lapse confocal microscopy of Fc:tdTom^+^ ECs after 28 days in culture revealed robust polarized MARCKS::YFP localization at the plasma membrane (Fig. S2; Movie S7). Upon PMA stimulation, MARCKS::YFP translocated to the cytosol and revealed elevation in localization to the membrane of intracellular vacuole-like structures throughout the cytosol (Fig. S2). This dissociation from the membrane and actin to cytosol following PMA stimulation was similarly noted in HBE1 cells (an established lung epithelial cell line) and validated by immunoprecipitation and Western blotting analysis of lysates harvested from HBE1 cells and ECs grown in culture (Fig. S2).

To examine MARCKS trafficking in adult ECs, we utilized acute wholemount preparations, as we are unable to culture adult ECs for extended periods of time. The MARCKS::YFP construct was electroporated into 2M and 2Y adult brains; wholemounts were harvested 60 hours post-electroporation and maintained *ex vivo* for up to 36 h. Time-lapse imaging of acute wholemount cultures revealed robust release of MARCKS from the membrane upon PMA treatment in young explants, whereas this dynamic response is far less consistent in old ependyma (Fig.1D–F; Movies S8–9). These findings demonstrate that phosphorylated MARCKS dissociates from the plasma membrane and concentrates on vacuole-like organelles in young ECs.

### MARCKS is required for Clca3 and mucin localization in ECs

**Fig 2:**
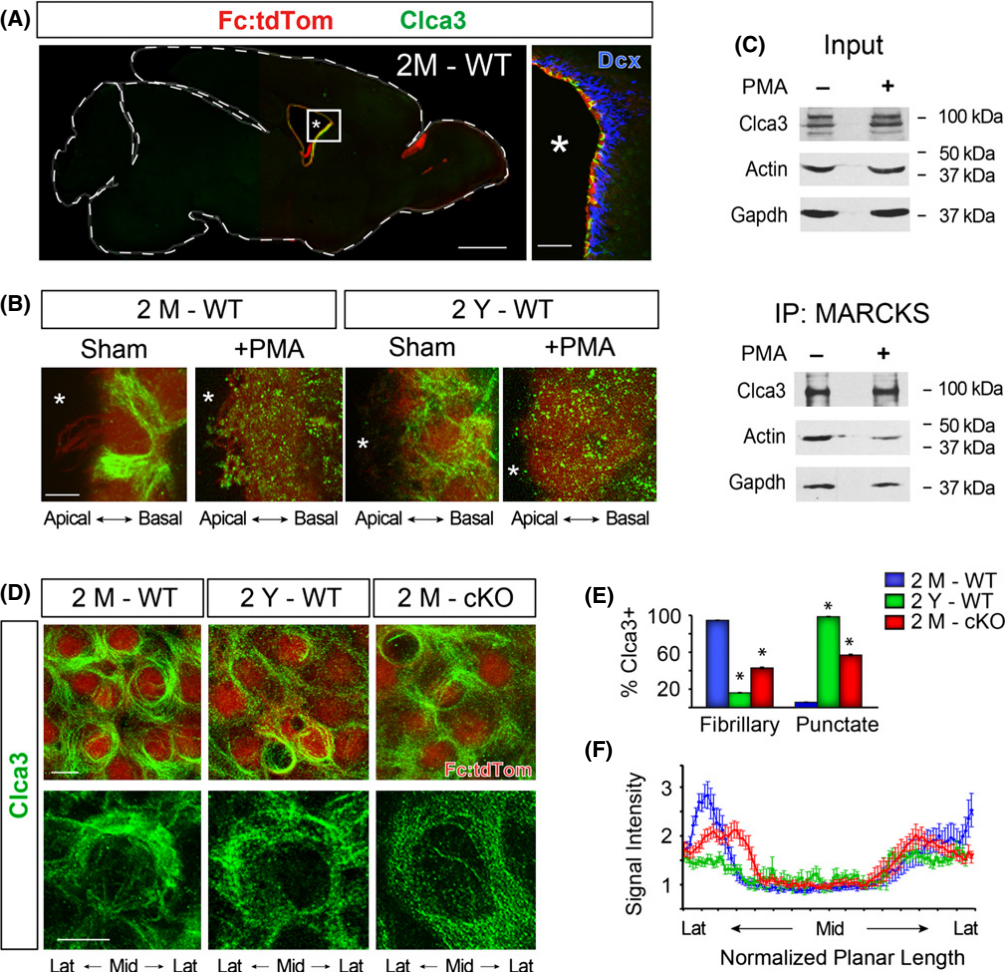
Clca3 expression in the forebrain is enriched in ECs, and its localization is dependent on expression of MARCKS and aging. (A) A sagittal brain section from a 2M Fc:tdTom mouse immunostained with a Clca3-specific antibody (green; tdTom+ ependyma, red; scale bar: 1 mm). Boxed area represents the zoomed image to the right showing colabeling with a doublecortin antibody (Dcx, blue) labeling subependymal progenitors and neuroblasts on the walls of the lateral ventricles (Scale bar: 50 μm). (B) High-magnification view of individual Clca3-stained (green) ependymal cells (red) following sham or PMA intraventricular injections prior to fixation five minutes later. Asterisks mark the ventricular lumen. (C) Western blots of Clca3, Actin, and Gapdh before (input) and after immunoprecipitation of MARCKS from ependymal wholemounts with (+) and without (-) PMA treatment. The difference in the number of bands in the ‘Input’ versus ‘MARCKS IP’ lanes may be due to differential posttranslational modified states of Clca3 in lysates versus when bound to MARCKS. Note that association between Clca3 and MARCKS is independent of PMA treatment. (D) High-magnification images of Fc:tdTom ECs (red) immunostained for Clca3 (green) in wholemount preparations from young (2M) and old (2Y) wild-type (WT) mice and 2M mice in which MARCKS was conditionally deleted in ECs (cKO). Scale bars: 5 μm. (E) Percentage of Clca3 +  ECs with fibrillary and punctate patterns of Clca3 distribution in 2M and 2Y WT and 2M MARCKS-cKO forebrains. Data are mean ± SEM; *n* = 3 animals per genotype and age; total of 300 cells per animal; *, Student’s *t*-test, *P* < 0.0001. (F) Quantification of signal intensity of Clca3 immunoreactivity in planar axis of ependymal cells. Data represent planar distribution normalized across planar length of ependymal cells away from midline (Mid) of individual cells toward their lateral (Lat) aspects. Data are mean ± SEM; *n* = 3 per age and genotype.

We next focused on defining the function of MARCKS in aging ECs. In lung epithelia which share numerous features with ependyma, MARCKS is postulated to regulate the trafficking and secretion of mucin granules (Park *et al*., 2007). Mucins are a family of glycoproteins that aid in barrier formation for a number of epithelial tissues lining fluid- or air-filled cavities of organs. In the lung, MARCKS is associated with mucin granules decorated with the protein chloride channel calcium-activated family member 3 (Clca3), and MARCKS phosphorylation influences levels of mucin secretion in the lung epithelia (Foster *et al*., 2010). Antibody labeling revealed that the expression of Clca3 in the forebrain is mostly confined to ECs (Fig.2A), and co-immunoprecipitation showed that MARCKS forms a complex with Clca3 in wholemount preparations (Fig.2C). To our knowledge, this is the first report of Clca3 expression in the brain with the majority of it localizing to ependymal cells.

High-magnification confocal imaging of Clca3-labeled cross sections and wholemounts revealed a ring-like distribution spanning the periphery of young ECs (Fig.2A, B). Unlike MARCKS, which has a polarized apical enrichment under wild-type conditions, Clca3 is concentrated near the basolateral junctions of ECs in regions of actin-rich membrane domains (Fig. S3). Moreover, α-tubulin is colocalized with Clca3 in domains partially overlapping with actin in ECs (Fig. S3). Although expressed in both 2M and 2Y ependyma, distribution of Clca3 in old ependyma is significantly more punctate and less fibrillary (fibrous) than in 2M WT brains (Fig.2B,D). To further correlate the impact of MARCKS phosphorylation on localization of Clca3 in ependyma, 2M and 2Y wild-type mice were intraventricularly injected with PMA followed by immediate perfusion and tissue analysis as described above. PMA-treated wild-type ependyma exhibit profound disruption of the tight Clca3 organization from a fibrillary pattern to punctate (Fig.2B).

To further examine the Clca3-MARCKS association in ECs, we deleted *MARCKS* in ependyma using a new mouse carrying its conditional alleles (*MARCKS*^*F/F*^; Fig. S4). Selectivity for MARCKS deletion to ECs was achieved by crossing the *MARCKS*^*F/F*^ mice to our Fc:tdTom line which expresses cre recombinase in ECs (Fig.1C; the *Fc:tdTom; MARCKS*^*F/F*^ genotype will be referred to as MARCKS-cKO, and Fc:tdTom/MARCKS^+/+^ as WT, hereafter; Fig. S4). High-magnification confocal imaging of wholemounts and brain sections revealed that Clca3 is scattered throughout the cytoplasm of MARCKS-cKO ependyma, unlike the tight fibrillary organization in 2M WT ependyma (Fig.2D). Quantitative assessment of planar distribution of Clca3 in ependyma revealed a significant disruption of its tight organization at 2M, in both 2Y and 2M MARCKS-cKO ECs (Figs.2D–F, S2). To confirm this finding using another approach, ECs cultured from MARCKS-cKO brains were transduced with a FOXJ1:Clca3::YFP encoding lentivirus followed by fixation. Imaging of these cells revealed a similar loss of fibrillary, ring-like Clca3 organization in MARCKS-cKO ECs compared to WT cultures (Fig. S5). Taken together, these findings demonstrate a highly organized MARCKS-dependent localization of Clca3 to actin/microtubule networks near the basal membranes of ECs.

**Fig 3:**
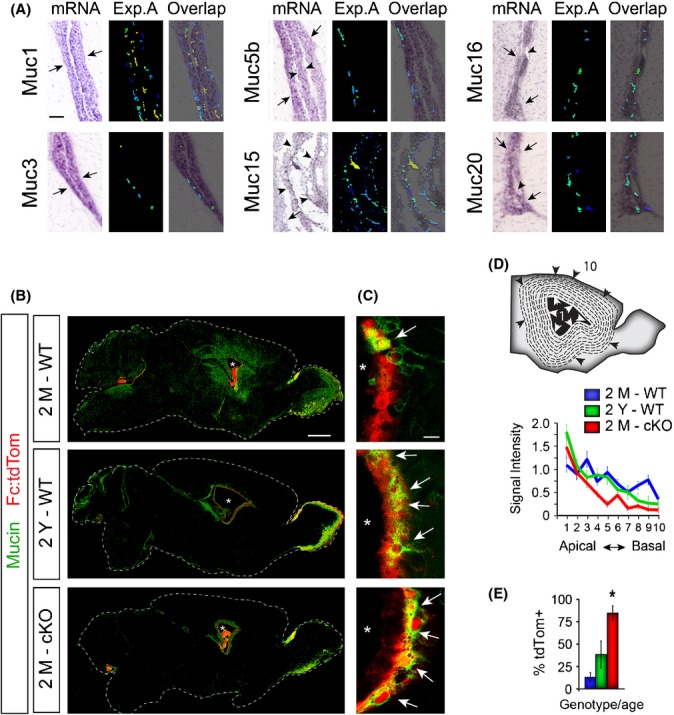
Conditional deletion of MARCKS exacerbates the natural age-associated accumulation of intracellular mucin in ECs. (A) Panels from the Allen Brain Atlas cropped to reveal the presence of mRNA (purple) for various mucin isoforms with confirmed expression in ECs (arrows) and choroid plexus (arrowheads). Expression analysis (Exp. A.) heat maps of the mRNA signal were obtained from the same resource and overlaid onto the mRNA image (Overlap). (B) Low-magnification deconvoluted images of confocal tile-scanned sagittal sections immunostained with a pan-mucin antibody (green). tdTom+ ECs are in red; scale bar: 500 μm. (C) High-magnification images of mucin stained ECs show significant increase in proportion of cells potentially retaining mucin in old WT and 2M MARCKS-cKO brains (arrows). Asterisks indicate the lumen of the ventricles. Scale bar: 10 μm. (D) Intensity indices of mucin immunoreactivity in the forebrain away from the ventricular zone (dotted concentric circles in cartoon 1-apical, 10-basal correspond to the gradient on the *x*-axis in chart). (E) Percentages of tdTom+ ECs colocalized with mucin immunoreactivity at various ages and in MARCKS-cKO brains. Data are mean ± SEM, *n* = 3 per age and genotype; *, Student’s *t*-test, *P* < 0.05.

The apparent MARCKS-dependent subcellular localization of Clca3 motivated us to focus on potential biological and physiological consequences of mislocalized Clca3 in MARCKS-cKO ECs. Clca3 is known to associate with mucin-containing granules in lung epithelia (Leverkoehne & Gruber, 2002). Although neither the presence nor the role of mucins in the ependymal lining has yet been explored, we hypothesized that mucins may be expressed and cleared by ependyma as they are in lung epithelial cells. A detailed screen of the Allen Brain Atlas revealed the presence of transcripts for multiple isoforms of mucins in the ependymal layer and the choroid plexus, with little to no expression in the interstitial compartments of the brain (Fig.3A). Immunostaining with a pan-mucin antibody revealed intermittent presence of mucin protein in only subsets of Fc:tdTom+ ECs in 2M WT mice (Fig.3B). Mucin+ ependyma are morphologically similar to juxtapositioned WT ECs, suggesting potential spatiotemporal regulation of mucin expression in ependyma.

Unlike selective presence of mucin transcripts in ECs, immunoreactivity for the mucin protein is distributed in a gradient within the interstitial tissue surrounding the ventricles of the 2M WT forebrain (Fig.3B, C). This suggested potential secretion and spread of mucins away from CSF-filled cerebral ventricles. The pattern of intermittent mucin-filled ependyma and graded distribution of mucin localization in the brain interstitium is dramatically altered in both 2Y WT and 2M MARCKS-cKO forebrains, where significantly higher proportions of tdTom+ ECs retain mucin intracellularly (Fig.3C, arrows; D,E). Taken together, these data demonstrate that expression and distribution of mucins in ECs and brain interstitium is both MARCKS and age dependent.

### Young MARCKS-null and old wild-type ECs contain elevated markers of oxidative stress and buildup of lipid droplets

It was recently demonstrated that oxidative stress induces phosphorylation of MARCKS, resulting in selective permeability of endothelial cells grown in culture (Jin *et al*., 2012). Thus, in the absence of MARCKS and p-MARCKS, responsiveness to oxidative stress may be defective, resulting in the elevation of oxidative state in cells. To assess whether oxidative state in ependyma changes with age and in the absence of MARCKS, we examined the levels of 4-HNE, which is an α,β-unsaturated hydroxyalkenal that is produced by lipid peroxidation and tyrosine nitration and is upregulated in instances of elevated reactive nitrogen species. Elevated levels of both 4-HNE and nitrotyrosine are significant in both 2M MARCKS-cKO and 2Y WT ependyma compared to their 2M WT counterparts (Fig. S6). Thus, disrupted Clca3 localization and buildup of mucins in ependyma are highly correlated with increased oxidative stress in these cells, a process that is sensitive to the expression of MARCKS.

**Fig 4:**
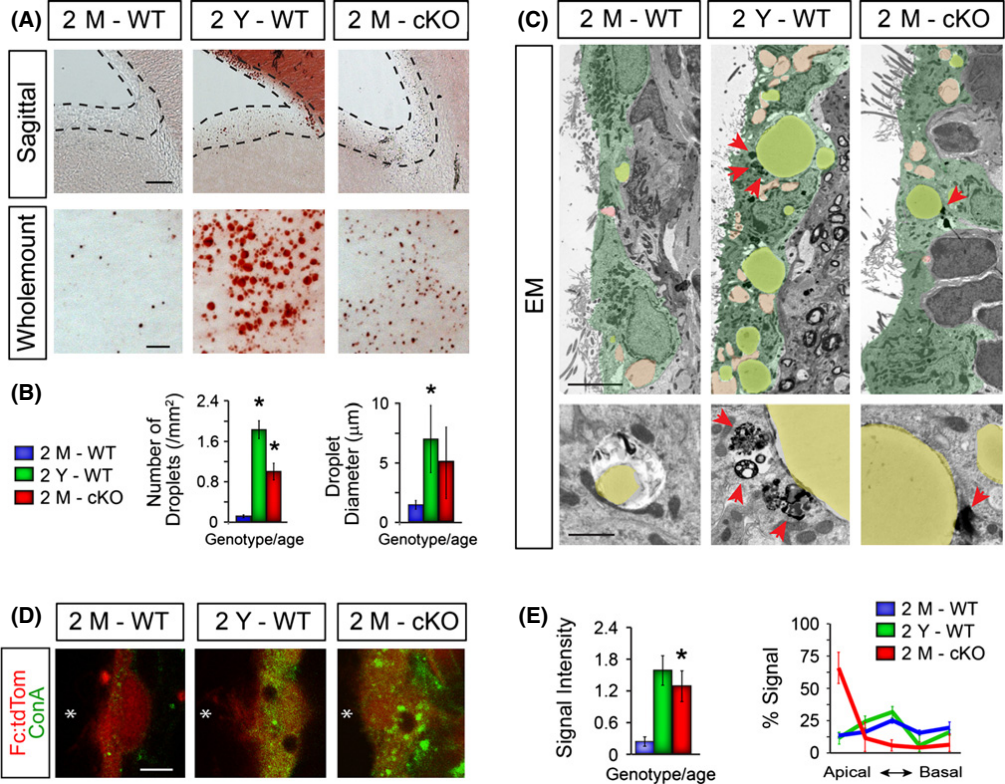
Conditional deletion of MARCKS alters lipid and carbohydrate metabolism in ECs. (A) Oil red O-stained sagittal sections and wholemounts across various ages and genotypes. Scale bars: sagittal, 50 μm; wholemount, 10 μm. (B) Quantification of density and diameter of lipid droplets in wholemount preparations. (C) Electron microscopy of young and old WT, and cKO ECs (green). Highlighted compartment: yellow, lipid droplets; pink, acellular gaps. Arrowheads point to lysosomal debris near large lipid droplets. Scale bars: 5 μm top row, 1 μm bottom row. (D) Immunostaining for concanavalin A (ConA, green; biomarker for carbohydrate metabolism) in ECs (Fc:tdTom, red). Asterisks indicate the lumen of the ventricles. Scale bar: 10 μm. (E) Quantifications of intensity, and apico-basal distribution of ConA immunoreactive signals in young, old, and MARCKS-cKO ependyma. Data are mean ± SEM, *n* = 3 per age and genotype; *, Student’s *t*-test, *P* < 0.05.

Another recent study reported that MARCKS influences membrane lipid composition and dynamics by regulating the membrane clustering of phosphatidylinositol-(4,5)-bisphosphate (PI(4,5)P₂) in neurons (Trovo *et al*., 2013). With age, neuronal synapses contain less membrane-associated MARCKS and presumably more of the internalized p-MARCKS. Based on these parallels, we reasoned that lipid composition or metabolism may be altered in the absence of MARCKS and in response to elevated oxidative stress and disrupted mucin clearance in ECs. Indeed, both the size and number of lipid droplets significantly increase in both young MARCKS-cKO and old WT ECs when compared to 2M WT mice (Fig.4A,B). Electron microscopy confirmed that lipid droplets observed in wholemounts and cross sections are intracellular in ECs and often accompany numerous lipid-free vacuoles and lysosomal debris (Fig.4C). Consistent with these results, we also found elevated levels and disrupted spatial distribution of the glucose-mannose-associated metabolic biomarker concanavalin A (ConA) in old ECs (Fig.4D,E). Importantly, precocious buildup of lipid droplets, disrupted ConA expression, and subcellular distribution were significant in MARCKS-cKO ECs by 2 months of age. Thus, the accumulation of mucins and lipid droplets is highly correlated with the significant increase in oxidative stress within both aged and MARCKS-cKO ECs.

### Conditional deletion of MARCKS results in disruption of barrier integrity in ECs and precocious elevation of astrogliosis and macrophage infiltration into the forebrain interstitium

**Fig 5:**
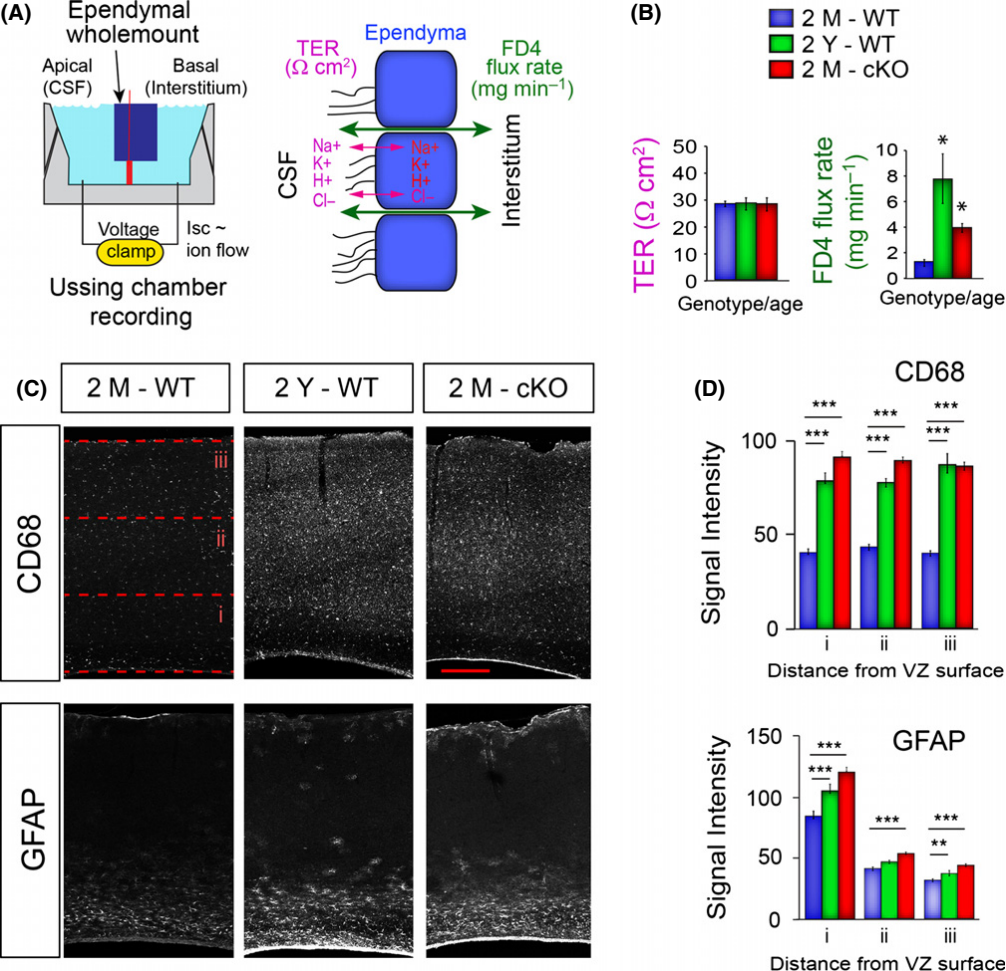
Compromised ependymal barrier function in MARCKS-cKO ependyma interrupts homeostasis in interstitial compartments of the forebrain. (A) Illustration of an Ussing chamber recording setup for measuring transepithelial current and pericellular flux in ependymal wholemounts. (B) Transepithelial resistance (TER, Ω cm^2^) and rate of FD4 flux (mg/min) as an indicator of paracellular barrier integrity, measured from young, old, and cKO wholemounts. Data are mean ± SEM, *n* = 3 per age and genotype; *, Student’s *t*-test, *P* < 0.05. (C) Immunostained sections of the cerebral cortex (ventricles are evident on the bottom of each micrograph) reveal CD68+ macrophage infiltration and GFAP+ astrogliosis in young, old, and cKO brains. Red dotted lines depict areas where measurements in (D) were quantified. Scale bar: 250 μm. (D) Quantification of CD68 and GFAP immunoreactive intensity measured from three equidistant bins from the ventricular toward the pial surfaces of the cortex (see red dotted lines in C). Data are mean ± SEM, *n* = 3 per age and genotype; *, Student’s *t*-test, *P* < 0.05; **, *P* < 0.01; ***, *P* < 0.001.

As neutral lipids are essential sources of energy for cellular function, we next hypothesized that age-associated lipid buildup, elevated oxidative stress, and the apparent disruption in mucin clearance combine to interfere with barrier functions in MARCKS-cKO and aged ependyma. Barrier integrity in the ependymal layer was tested by measuring transepithelial electrical resistance (TER) and by performing a FITC–dextran (FD4) assay in wholemounts using Ussing chambers (Fig.5A). TER, a measurement of paracellular conductance of ions, is not influenced by age or MARCKS deletion in ependyma (Fig.5B). In contrast, FD4 flux rate, a measure of paracellular permeability to high molecular weight substances, is significantly elevated in 2Y WT compared to 2M WT wholemounts, indicating loss of ependymal barrier integrity with aging (Fig.5B). Remarkably, 2M MARCKS-cKO ependymal wholemounts exhibit a loss in barrier function similar to 2Y WT brains, supporting the hypothesis that the deletion of MARCKS precociously ages ECs in the context of lipid metabolism, mucin distribution, and barrier functions.

**Fig 6:**
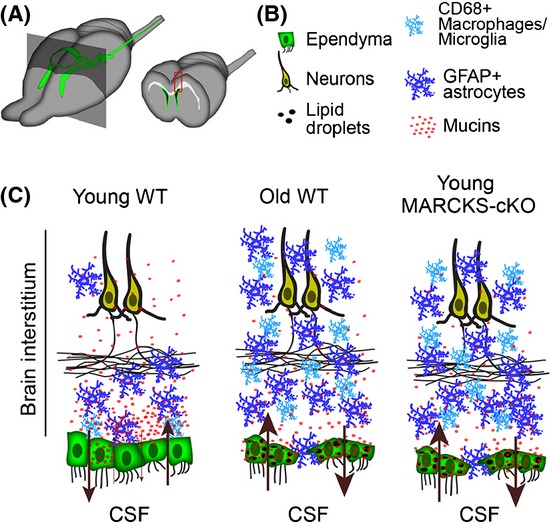
Working model of the role for ependyma in maintenance of homeostasis in the forebrain during aging. (A) Ventricular system of the mouse forebrain (green) is lined with ECs. Coronal view through the forebrain reveals the layers of fluids and tissue in the aging brain; CSF in the ventricles (black), ECs (Green), interstitial compartments (white and gray). Constituents in red boxed area in the coronal view are depicted in (C). (B) Key for colored constituents in (C). (C) Cellular composition within interstitial compartments is disrupted in young MARCKS-cKO and old WT compared to young WT mice. Mucins appear retained in ependyma with loss of their gradient in the brain interstitium in young MARCKS-cKO and old WT brains. This is in conjunction with the accumulation of lipid droplets in ECs. Brown arrows indicate controlled and largely unleaky nature of the ependymal barrier in the young WT versus its collapse in the young MARCKS-cKO and old WT brain. Apparent precocious aging of young ependyma in response to loss of MARCKS results in astrocytic, microglia, and macrophage infiltration (blue cells) into the interstitial layers as in normal aging, indicative of possible increase in reactive astrogliosis and macrophage accumulation in the central nervous system.

Finally, the impact of precocious aging of MARCKS-cKO ECs on potential gliosis and neuroimmune responses in interstitial brain tissue was assessed. First, effects of normal aging on the integrity of the ependymal layer in the forebrain were documented in wholemount preparations of the ventricular walls in young and old brains. Imaging clearly illuminated scarring of ECs accompanied by astrocyte infiltration into the scarred regions (Fig. S7). These periventricular defects in the 2M MARCKS-cKO brain are highly analogous to the 2Y wild-type mice. Additionally, MARCKS-cKO mice exhibit significant elevation in microglia and macrophages (CD68+ cells) as well as reactive astrogliosis (GFAP+ cells) in their cerebral cortex, again phenocopying defects in the cortex of aged WT mice (Fig.5C,D). These findings indicate that selective deletion of MARCKS in the monolayer of ECs results in reactive astrogliosis, as well as microglia and macrophage infiltration into the cerebral cortices. Interestingly, these defects are similar to disruption of mucin distribution and ependymal paracelluar barrier integrity in 2Y WT ECs (Fig.6).

## Discussion

### Potential role of MARCKS in known EC functions

MARCKS-dependent mechanisms used as a model in our study likely represent one of many that can influence aging and maintenance of ECs. ECs have been credited with multiple functional attributes which are possibly influenced by MARCKS-dependent cellular and molecular mechanisms. For example, a unique population of ependyma in the choroid plexus is responsible for the production of CSF and various transport proteins (Montecinos *et al*., 2005). Also, ECs that line the wall of the lateral ventricles provide trophic support to subependymal zone stem and progenitor cells (Lim *et al*., 2000; Jacquet *et al*., 2011). Moreover, motile cilia on the apical/luminal surface of ependyma display a directional lashing stroke which helps steer the flow of CSF within the ventricles (Sawamoto *et al*., 2006). It is therefore of no surprise that ependymal cell dysfunction has been increasingly implicated in neurological diseases through altered ciliary motility, breakdown in barrier function, and/or failure to properly produce, secrete, and filter the CSF.

Whether the stroke capacity of motile cilia in MARCKS-null ependyma is affected remains to be determined. The slight increase in ventricular volume in MARCKS-null mice (unpublished observations) may be indicative of a potential role for MARCKS on biomechanical properties of stroke in EC cilia. Indeed, the apical clustering of MARCKS near the base of EC cilia is suggestive of potential interaction with molecular motors that facilitate the beating of motile cilia. Ependyma in the young adult brain have been described to be heterogeneous based on their multiciliated or biciliated morphology (Mirzadeh *et al*., 2008). Moreover, we observed numerous unciliated ependyma in the old ventricular zone in this study, which has also been reported previously (Luo *et al*., 2008). As we did not distinguish among these EC types, it will be interesting to determine the distribution patterns of MARCKS, p-MARCKS, and aPKC_ζ_ in the different types of ECs based on their ciliary status.

While mechanical functions of motile cilia in ependyma are unequivocal, whether they perform any sensory functions similar to primary cilia remains for the most part unknown. Primary cilia have emerged as important sensors of circulating proteins in the CSF (Guemez-Gamboa *et al*., 2014). Interestingly, motile cilia in the lung epithelium harbor proteins such as serum response factor which have assigned sensory functions to these organelles (Nordgren *et al*., 2014)—whether or not such sensory functions are also present in EC motile cilia remains to be determined.

The bidirectional barrier and transport system provided by the ependymal lining of the brain have been largely postulated to assist in CSF-interstitial fluid exchanges that help keep the brain toxin free and in physiologic balance (Del Bigio, 2010; Roales-Bujan *et al*., 2012). Age-associated ventriculomegaly is one of the most common symptoms of ependymal dysfunction and is often attributed to neurodegeneration. However, additional factors such as compromise in ependymal barrier integrity could possibly initiate or contribute to progressive age-associated functional decline in the brain or serve as an instigator of various neurological diseases. In the present study, a significant age-dependent elevation in permeability across the ependymal barrier was recorded from 2 months to 2 years of age in mice, but transepithelial resistance (TER) remained unchanged. The conflicting data between paracellular FD4 flux and TER have been observed by others (Santos & Perdue, 2000) and likely reflect different epithelial properties measured by TER versus FD4 flux. For example, TER is largely reflective of the apico-basal transcellular flux of small ions, whereas FD4 flux is a measure of paracellular flow of large molecules. In fact, IL-13-dependent upregulation of the tight junction protein claudin-2 was shown to increase paracellular permeability to cation flux without altering tight junction size selectivity (Marchiando *et al*., 2010; Weber *et al*., 2010). These ‘leak’ and ‘pore’ pathways, respectively, suggest differential regulation of paracellular permeability pathways (Weber *et al*., 2010; Shen *et al*., 2011) and could explain the present data.

### Potential role of MARCKS in the regulation of mucin trafficking and lipid metabolism in ECs

Our study for the first time reveals a novel role for the membrane-associated protein MARCKS in the localization of mucins and Clca3 and potential regulation of lipid droplet formation in ECs. Surprisingly, these processes appear to be negatively regulated during aging in ependyma of wild-type mice, in a MARCKS-dependent manner. Our findings suggest that mucin is likely secreted into the brain interstitium, but mucin secretion, distribution, and function remain to be determined. Nevertheless, we can glean some understanding from the function of mucins in other cell types. For example, mucins are important constituents of mucus in the lung, leading us to speculate that a mucus-like secretion and spread from ECs may provide lubrication in yet-to-be defined corridors in the brain parenchyma. Additionally, mucins may provide protection against infection in the brain similar to their role in the respiratory tract. Our analysis of the mucin-associated protein Clca3 in ECs will be useful in future studies of mucin physiology in the brain.

Our study for the first time reports the expression of Clca3 in the brain with high specificity to ependymal cells. Clca3 (also known as GOB5) was originally identified in intestinal goblet cells and gained attention for its selective expression in the lung epithelium and its role in mucus secretion during airway hyper-responsiveness (Nakanishi *et al*., 2001). Although Clca3 belongs to a family of transmembrane chloride channels, its participation in chloride flux is debated. For example, Clca3 does not integrate into the membrane and itself may be secreted (Gibson *et al*., 2005; Loewen & Forsyth, 2005). Here, we found that the planar distribution of Clca3 was restricted to areas adjacent to the basolateral membranes of ECs. This pattern may suggest mucin secretion into the brain parenchyma from these junctional domains in young ependymal cells. In contrast, more diffuse pattern of Clca3 and its mislocalization may account for potential defects in mucin secretion, which may underlie the intracellular accumulation of mucin in old WT and young MARCKS-cKO ECs. However, direct involvement of Clca3 in mucin trafficking and secretion from ECs are yet to be determined. We discovered that Clca3 is present in nearly all ECs, whereas mucin is intermittently present in scattered young ependyma. This pattern suggests either mucins are rapidly cleared from ECs or Clca3 has additional functions to a role in mucin granule decoration. Our finding that a significantly larger fraction of young MARCKS-cKO and old wild-type ECs contain mucin than young wild-type ependyma is supportive of a model in which mucins are cleared from ependyma and that this capacity is diminished during aging.

Ependyma have been shown to accumulate lipids in the aged mouse brain (Bouab *et al*., 2011). Our current findings suggest that elevated lipid accumulation discovered in 2-month-old MARCKS-cKO ECs is similar to changes that occur in aging wild-type ependyma and may be related to disrupted mucin trafficking. Mucins are major components of mucus which has been shown to regulate lipid trafficking and metabolism in several cell types. For example, human gallbladder mucins bind to cholesterol-filled vesicles, resulting in vesicle aggregation and eventual increase in size (Afdhal *et al*., 2004; Wang *et al*., 2006). Overexpression studies of the mucin-1 intracellular domain have suggested that mucins also play an upstream role in the regulation of metabolic genes (Pitroda *et al*., 2009), some of which directly affect synthesis of cholesterol, fatty acids, and triglycerides (Horton *et al*., 2002). However, whether lipid droplet accumulation in ECs is due to enhanced uptake of lipids from the CSF or overlying neural tissues, or is a result from disrupted metabolism in ECs remains to be tested. Given that recent studies have revealed the presence of early endosomes in apical domains of ependymal cells (Roales-Bujan *et al*., 2012), uptake of lipids may be a plausible source for the observed accumulation of lipid droplets with age in ECs.

We also found elevation of 4-HNE and nitrotyrosine in ECs of MARCKS-cKO mice similar to aged wild-type brains. 4-HNE is an α,β-unsaturated hydroxyalkenal that is produced by lipid peroxidation and tyrosine nitration. It is upregulated in instances of elevated reactive nitrogen species and, together with nitrotyrosine, is routinely used as a biomarker for oxidative stress. Interestingly, elevated intracellular oxidative stress has been demonstrated to lead to induction of lipid droplet formation and fatty acid accumulation in several cell types (Kuramoto *et al*., 2012). Thus, buildup of lipid droplets and the significant decline in barrier capacity of young MARCKS-cKO and old wild-type ependyma may be due to elevated oxidative stress in ECs. While we can only speculate regarding the causes of increased oxidative stress in ependyma during normal aging, our results suggest that MARCKS-dependent mechanisms are required for protection against oxidative damage in ependyma of the young brain.

Our findings have revealed that selective disruption of barrier functions in ECs can age the interstitial brain tissue (Fig.6). This paradigm is a departure from the prevailing view that age-associated changes in ependymal and periventricular brain tissues are secondary to aging of the overlying interstitium. Although we have little information regarding the aging of ependymal cells in the human brain, a recent study provides a link between ventricular surface gliosis and ventriculomegaly (Shook *et al*., 2014). The physiological significance of ependyma-integrated astrocytes, which are the main components of ventricular surface gliosis, remains to be explored. Glial expansion into the EC layer during aging could be protective as these astrocytes may attempt to reestablish barrier integrity in aged ependyma by forming junctional complexes with neighboring ECs (Luo *et al*., 2008; Roales-Bujan *et al*., 2012).

However, ependyma-infiltrated astrocytes may contribute toward elevated oxidative damage in periventricular tissues. For example, it was recently demonstrated that age-associated increase in mitochondrial metabolism in astrocytes produces detrimental reactive oxygen and nitrogen species (Jiang & Cadenas, 2014). These age-dependent metabolic changes were reproduced in young astrocytes upon exposure to inflammatory cytokines (IL-1β and TNF-α), which may result in neuroinflammatory and degenerative responses. Alternatively, the elevated GFAP signal documented in the ependymal layer of aged and young MARCKS-cKO mice may be due to increase in astrocytic processes of adult and aging stem cells that are in contact with the ventricles and nearby blood vessels (Doetsch *et al*., 1999; Mirzadeh *et al*., 2008). Future studies will be required to dissect the mechanisms for the integration of astrocytes in the ependymal surface and identifying their role in the EC layer.

In summary, our study demonstrates for the first time that MARCKS is required for maintaining the ependymal barrier integrity and sustaining forebrain homeostasis during normal aging. In addition, we have identified that mucins are expressed by, and may be secreted from, ependymal cells into the brain interstitium in an age- and MARCKS-dependent manner. These functions are correlated with lipid droplet formation in ependyma raising the possibility that MARCKS-dependent mucin expression and distribution in the brain parenchyma may be related to lipid metabolism in aging ECs. Thus, conditional MARCKS deletion in ependyma together with the apparent precocious aging of ECs provides a highly suitable *in vivo* model for assaying the role of ECs in maintenance of brain homeostasis under various disease states and environmental stimuli with potential relevance to human aging.

## Experimental procedures

Experimental Procedures are available in Supporting information.

## Supporting Information

**Fig. S1.** Individual channels from Figure B. **Fig. S2.** Conserved dynamics of MARCKS in ependymal cells and HBE1 lung epethilial cells. **Fig. S3.** Overlap of Clca3 with actin and microtubule networks in ependymal cells. **Fig. S4.** Generation and characterization of conditional (floxed) MARCKS mice. **Fig. S5.** Distribution of Ccla3::YFP in cultured ependymal cells. **Fig. S6.** Distribution of oxidative stress markers 4HNE and Nitrotyrosine in WT and MARCKS-cKO ependymal cells. **Fig. S7.** Astrocyte infiltration into the ependymal walls of aged and MARCKS-cKO forebrains.

**Appendix S1.** Experimental Procedures.

**Movie S1.** Movie of confocal Z-stacks scanned in sagittal brain slices immunostained for MARCKS (red) of 2M-WT ECs (green) before and after PMA treatment.

**Movie S2.** Movie of confocal Z-stacks scanned in sagittal brain slices immunostained for p-MARCKS (red) in 2M-WT ECs (green) before and after PMA treatment.

**Movie S3.** Movie of confocal Z-stacks scanned in sagittal brain slices immunostained for aPKC (red) in 2M-WT ECs (green) before and after PMA treatment.

**Movie S4.** Movie of confocal Z-stacks scanned in sagittal brain slices immunostained for MARCKS (red) of 2Y-WT ECs (green) before and after PMA treatment.

**Movie S5.** Movie of confocal Z-stacks scanned in sagittal brain slices immunostained for p-MARCKS (red) in 2Y-WT ECs (green) before and after PMA treatment.

**Movie S6.** Movie of confocal Z-stacks scanned in sagittal brain slices immunostained for aPKC (red) in 2Y-WT ECs (green) before and after PMA treatment.

**Movie S7.** Time-lapse confocal imaging of Fc:tdTom+ ECs (red) cultured for 28 days after electroporation with a MARCKS::YFP construct. MARCKS::YFP (green) robustly associates with the membrane of ECs and appears unaffected upon vehicle (DMSO) treatment. Addition of PMA to the culture medium immediately stimulates dissociation of MARCKS from the membrane and its intracellular transport to vacuole-like structures (round organelles) in ECs.

**Movie S8.** Time-lapse imaging of an acute 2M-WT ependymal wholemount.

**Movie S9.** Time-lapse imaging of an acute 2Y-WT ependymal wholemount preparation maintained *ex vivo*, and expressing MARCKS::YFP (gray) before and after PMA treatment.
