# Supplementary material for: MARCKS-dependent mucin clearance and lipid metabolism in ependymal cells are required for maintenance of forebrain homeostasis during aging
Source: Aging Cell. 2015 May 25;14(5):764–73. doi: 10.1111/acel.12354 (PMC4568964; doi:10.1111/acel.12354)
Supplement: Supplementary file 1 [file acel0014-0764-sd1.pdf]

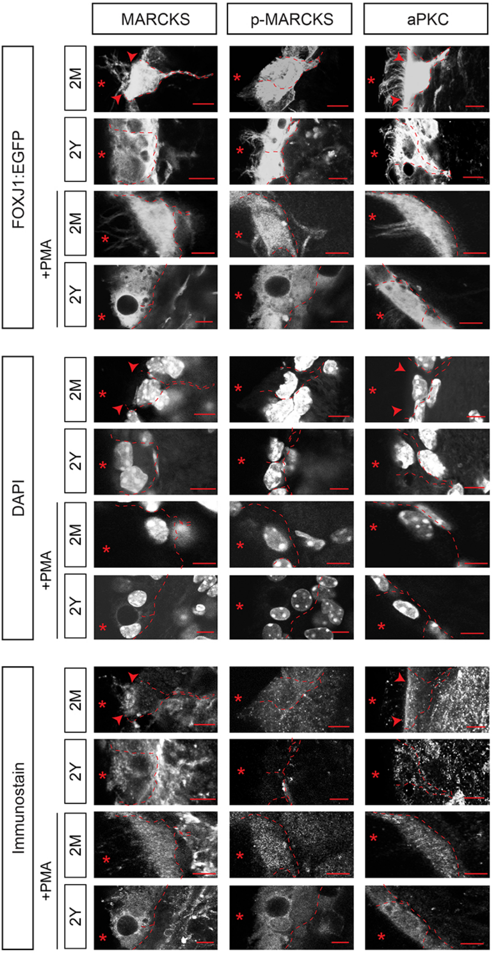

Supplementary Figure 1. Individual channels from Figure 1B. FOXJ1:EGFP transgenic mice with EGFP labeled ECs (top panel, outlined with red dotted lines) were utilized for immunofluorescence analysis. Middle panel illustrates nuclei labeled with DAPI in the same images. Bottom panel is immunoflurescence signals for MARCKS (left column), phosphorylated MARCKS (p-MARCKS, right column) and atypical PKC zeta (aPKCζ, right column) in young (2M) and old (2Y) brains. "+PMA" marks sections obtained from 2M and 2Y FOXJ1:EGFP brains which were intraventricularly injected with PMA and perfused 5 minutes later. Asterisks indicate the lumen of the ventricles. Scale bars: 10 µm.

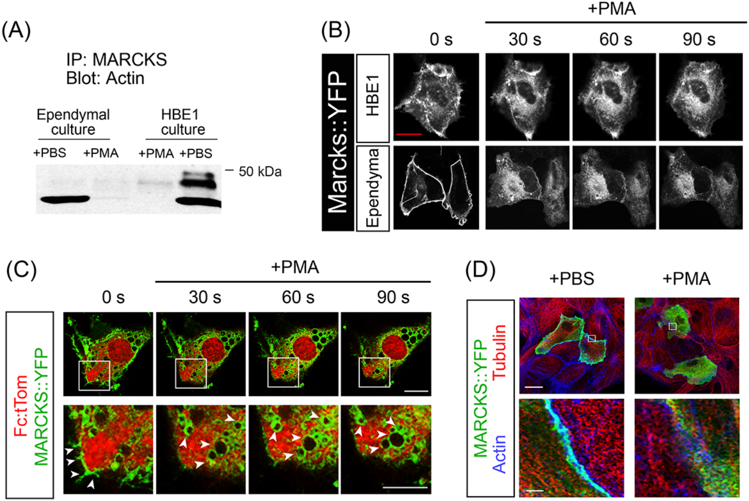

Supplementary Figure 2. Conserved dynamics of MARCKS in ependymal cells and HBE1 lung epethilial cells. **(A)** HBE1 lung epithelium and ependymal cells in culture with and without PMA treatment were lysed, immunoprecipitated for MARCKS, followed by blotting for actin. **(B)** Time-lapse imaging of HBE1 and ependymal cells expressing MARCKS::YFP before and after PMA treatment (+PMA). Scale Bar: 5 µm. **(C)** Time lapse panels of Fc:tdTom ependymal cells (red) electroporated with a MARCKS::YFP expression plasmid (green) in culture and acutely stimulated with PMA. PMA induced dissociation of MARCKS::YFP from the plasma membrane into internal vacuole-like structure (arrowheads). Bottom row are zoomed panels of area boxed in the top row. Scale bars: top row, 10 µm; bottom row, 5 µm. **(D)** Immunostaining for actin (blue) and tubulin (red) in HBE1 cells treated with PBS or PMA. Boxed areas in ltp panels are zoomed in the bottom panels. Scale Bars: low magnification, 5 µm; high magnification, 0.5 µm.

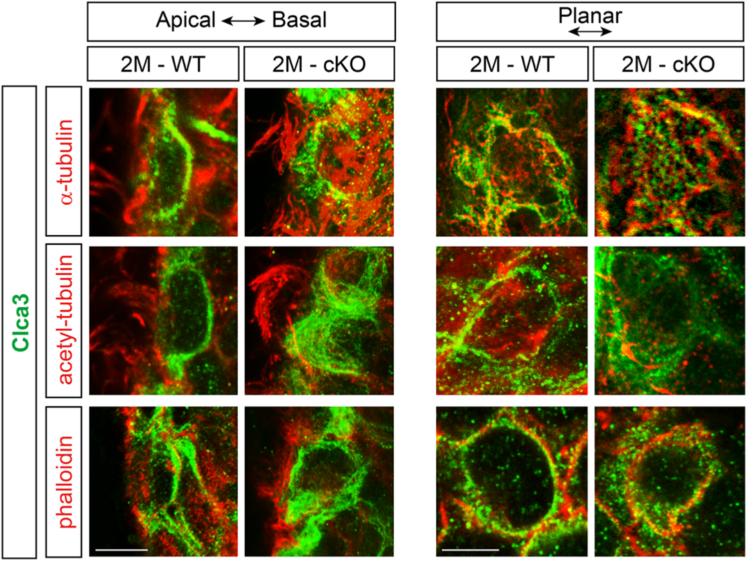

Supplementary Figure 3. Overlap of Clca3 with actin and microtubule networks in ependymal cells. Confocal micrographs of individual ependymal cells in sagittal sections (apical-basal view) and wholemount prepartions (planar view) from 2 months old WT and MARCKS-cKO mice. Tissue was labeled with Clca3 (green) together with markers for tubulins (α-tubulin, acetyl-tubulin, red) and F-actin (phalloidin, red). Scale bars: apical-basal, 10 mm; planar, 5 mm

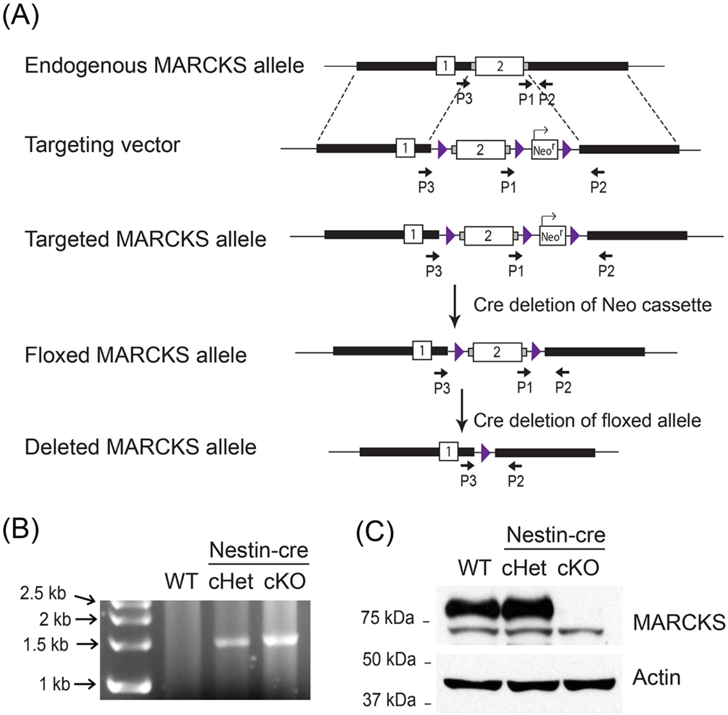

Supplementary Figure 4. Generation and characterization of conditonal (floxed) MARCKS mice. (A) Map and flow chart for generation of targeting vector and cre-lox mediated deleiton of MARCKS. Description is provided in Experimental Procedures. White boxes, exons; Black boxes, introns; Closed purple triangles, loxP sites; P1-P2, PCR primers used to genotype floxed mice; P3 and P2 primers were used for genomic detection of deleted MARCKS allele. Neo^r, neo resistance cassette for selection. **(B-C)** Characterization of deletion of the floxed MARCKS allele using a Nestin-cre transgene with highly sufficient recombination in the brain during perinatal development. P21 Nestin-cre; MARCKS floxed mice were harvested for either genomic (B) or protein (C) analysis. Pimers P3 and P2 were used to detect deleted alleles by PCR. The wildtype band using these primers results in a product greater than 4 kb which is not present in this picture. Western Blotting confirmed robust deletion of MARCKS in cKO brains compared to cHet and WT brains. Actin blotting served as loading control.

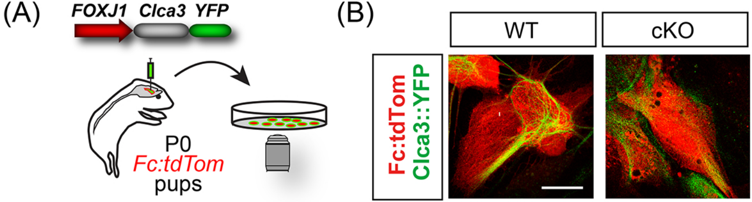

Supplementary Figure 5. Distribution of Clca3::YFP in cultured ependymal cells. (A) A Clca3::YFP fusion cassette downstream of the FOXJ1 promoter was cloned into a replication incompetent vector derived from the equine infectious anemia virus (EIAV) for selective transduction in ECs (Jacquet et al., 2009a). EIAV-FOXJ1:Clca3:EYFP was injected into the lateral ventricles of P0 wildtype (WT) and MARCKS-cKO (cKO) mice on the Fc:tdTom background and harvested ependyma were cultured from injected brains two hours post injection and cultured for 28 days. **(B)** EIAV-FOXJ1:Clca3::EYFP transduced Fc:tdTom WT and MARCKS-cKO ECs after 21 days in vitro. Scale bar: 10 µm.

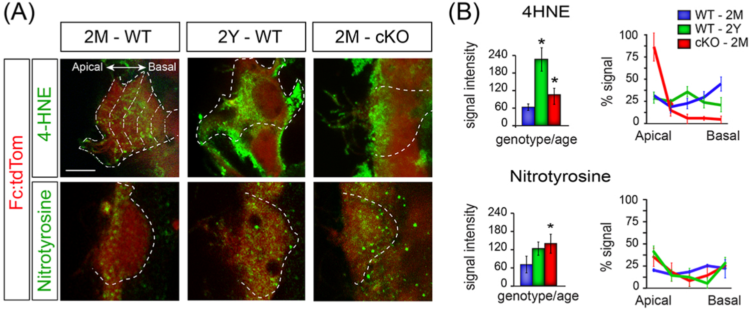

Supplementary Figure 6. Distribution of oxidative stress markers 4HNE and Nitrotyrosine in WT and MARCKS-cKO ependymal cells. (A) Confocal micrographs of individual ependymal cells in sagittal sections (apical-basal view) from 2M and 2Y WT and 2M MARCKS-cKO mice. Tissue was labeled with respective oxidative markers (green) overlayed with the Fc:tdTom signal from ependymal cells. Parallel demarcations in the first panel represent bins in which signal intensities in the apical-basal axis of ECs were measured throughout the study. Scale bar: 10 μm. **(B)** Quantification of overall signal intensity (bar charts) and apico-basal gradient of signal (line charts) in individual ependymal cells. Data in bar charts are average intensities, while data in line charts are percentage of signal within 5 apico-basal equidistant grids as dipicted in (A) 2M-WT, 4-HNE panel. Data are mean +/- s.e.m (n=30 cells from each of 3 animals); *, $p < 0.05$ Student's t-test.

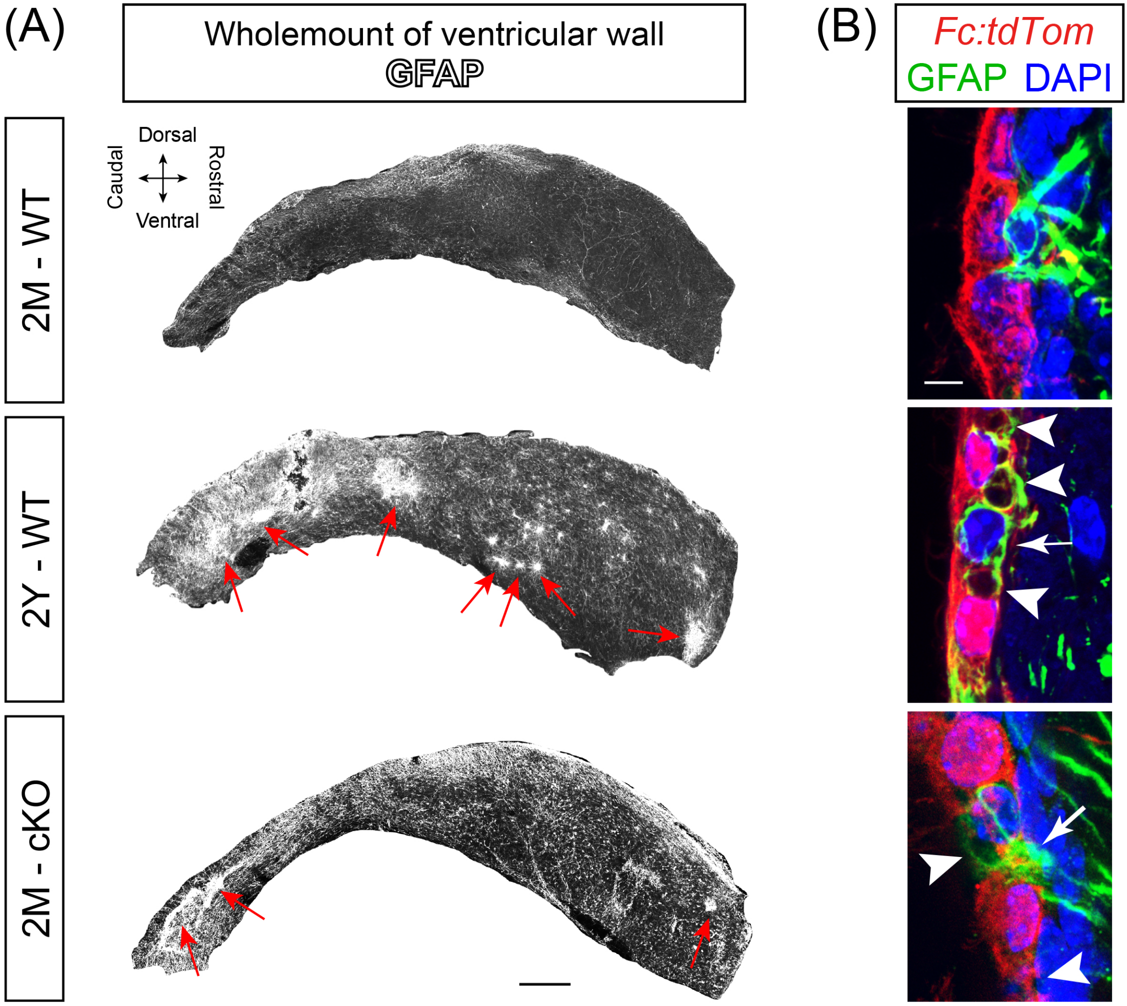

Supplementary Figure 7. Astrocyte infiltration into the ependymal walls of aged and MARCKS-cKO forebrains. (A) Wholemounts of ventricular walls from 2M-WT, 2M-cKO, and 2Y-WT forebrains stained with the reactive astrocyte marker GFAP. Red arrows point to areas with intense GFAP+ immunoreactivity. Scale bar: 500 μm. **(B)** High magnification imaging details the ependymal localization of astrocytes in the 2M MARCKS-cKO, 2Y-WT, 2M-WT periventricular zones. Arrowheads point to vacule buildup in the 2Y-WT and MARCKS-cKO ependymal layer. Scale bar: 5 μm.
