# Supplementary material for: MARCKS-dependent mucin clearance and lipid metabolism in ependymal cells are required for maintenance of forebrain homeostasis during aging
Source: Aging Cell. 2015 May 25;14(5):764–73. doi: 10.1111/acel.12354 (PMC4568964; doi:10.1111/acel.12354)
Supplement: Supplementary file 2 [file acel0014-0764-sd2.pdf]

Supporting Information

Experimental Procedures

Animals

All animals were maintained at animal facilities at North Carolina State University and Sanford Research according to guidelines from Institutional Animal Care and Use Committees. The following mouse strains were used for visualizing ECs *in vivo*: FOXJ1:EGFP mice to label ependyma green (Jacquet et al., 2009b), and FOXJ1:Cre mice on a tdTomato (tdTom) reporter background [*B6;129S6Gt(ROSA)26Sor*$^{tm14(CAG\text{-}tdTomato)Hze}$*/J*; Jackson Laboratory Stock: 007908; referred to as Fc:tdTom mice throughout the manuscript (Jacquet et al., 2011)]. Heterozygous mice with 'floxed' *MARCKS* allele (*MARCKS*$^{F/+}$) were generated by Xenogen Biosciences (Cranbury, NJ) using standard embryonic stem cell gene targeting techniques (See below). *MARCKS*$^{F/F}$ mice were bred to Fc:tdTom mice to selectively delete MARCKS in ependyma. All animals were backcrossed onto the C57BL/6J background during expansion of each colony, and housed on 12 hour light–dark cycle with ad libitum access to food and water.

Generation of MARCKS conditional mice

A 5.7 kb *NotI*/*SfiI* 5' homologous arm, a 4.9 kb *AscI*/*NruI* 3' homologous arm, and a 2.4 kb *BsiWI*/*SalI* region (cKO) were generated from the 193C14 BAC clone using recombineering methods. The 5' homologous arm and the cKO region were subsequently subcloned into 3loxP3NwCD and the 3' homologous arm was subcloned into pcR2.1. The final targeting vector was obtained by standard

molecular cloning and contained loxP sequences flanking the cKO region (designed to delete exon 2, including the polyadenylation signal). A loxP flanked Neo^r expression cassette was inserted between the cKO region and the 3' homologous arm which allowed for subsequent positive selection in ES cells. A Diphtheria toxin A (DTA) cassette was also present for negative selection of ES cells. The final targeting vector was confirmed by restriction digestion and end-sequencing. C57BL/6 ES cells were electroporated with 30 μg of *NotI* linearized targeting vector and Neo^r clones were selected with 200 μg/ml G418. Primary screening for homologously recombined clones was done by Southern analysis using a 5' external probe. Six clones were selected for expansion as potential positive clones. Subsequent Southern analysis using a 3' external probe resulted in 4 correctly targeted clones all of which contained a single Neo^r insertion based on TaqMan PCR analysis as well as a third loxP site. *Cre*-mediated deletion of the Neo cassette was performed by transient transfection in two positive ES clones. Deletion of the Neo^r cassette was confirmed by PCR analysis. Two independent, correctly targeted, Neo^r-deleted ES clones were injected into blastocysts to generate chimeric mice. Chimeric mice were mated to C57BL/6NTac mice to generate heterozygous floxed offspring. Offspring were routinely genotyped by PCR using primer 1 (P1), 5'-ATTTGGCTGTGGTTTTTGATAAGAAGTT-3', and primer 2 (P2), 5'-CCAAACTGACAATAAGTCCATTCAACACC-3'. These primers amplified a 404 bp endogenous, wild type (WT) allele, and a 574 bp floxed *MARCKS* allele ($MARCKS^{F/F}$). $MARCKS^{F/F}$ mice were first bred to Nestin-cre mice (Jackson Labs Bar Harbor, stock# 003771), expressing cre recombinase under the

control of the Nestin promoter. Brains were harvested for DNA and protein lysates preparation to demonstrate the deletion of MARCKS in tissue co-expressing cre-recombinase (Fig. S3, Supporting information).

Clca3 Lentiviral Production

Full-length mouse Clca3 tagged to Yellow Fluorescent Protein (Clca3::YFP; gift of Dr. Lars Mundhenk Freie Universitaet Berlin, Germany) was subcloned into a vector derived from the replication incompetent equine infectious anemia virus (EIAV) which is highly suitable for selective transduction in ependymal cells (Jacquet et al., 2009a). Clca3 lentivirus was produced by triple transfection of HEK293 cells as previously described (Jacquet et al., 2009a).

Acute- and Long- term Ependymal Cultures and Time-lapse Imaging

For acute in vivo experiments the MARCKS::YFP construct (Fang et al., 2013) was electroporated into adult brains (2 months and 2 years old Fc:tdTom mice) following procedures described before (Barnabe-Heider et al., 2008). Briefly, 20 µg of the construct was stereotaxically injected into the lateral ventricle of anesthetized mice; 1 mm lateral and 2.5 mm deep at the Bregma using manually guided Hamilton microliter syringes. The syringe was slowly retracted after 5 minutes of injection and mice were electroporated by delivering five 50 ms pulses of 200 V at 950 ms interval to the lateral surface of the skull. Following electroporation, mice were allowed to recover for 48 hours and their brains were harvested for wholemount cultures as described before (Mirzadeh et al., 2010).

Wholemounts were live imaged on a glass bottom dish using an air 40x objective and an Olympus confocal microscope equipped with temperature and CO_2 control. PMA was administered to culture media at a final concentration of 200 nM and live imaging was continued.

For long term experiments the EIAV-FOXJ1:Clca3::YFP lentivirus was injected into the lateral ventricles of MARCKS-cKO and control mice at P0. Mice were sacrificed 24 hours post injection, and ECs were cultured as previously described (El Zein et al., 2009; Guirao et al., 2010). The timing of electroporation (P0) was selected as radial glial cells in the ventricular zone begin their differentiation into ECs perinatally (Jacquet et al., 2009b) and are highly suitable for culturing at this age.

Intraventricular injections of phorbol-12 myristate-13 acetate (PMA)

PMA (4 µl, 4 µM in DMSO) or Sham (DMSO) was injected into the lateral ventricle of anesthetized mice (375 µg avertin/ gram body weight) using manually guided Hamilton microliter syringes and standard stereotactic procedures (coordinates: 1 mm lateral and 2.5 mm deep at the Bregma). After 5 minutes, PMA injected mice were transcardially perfused with 4% paraformaldehyde (PFA) and brains were removed and post-fixed overnight in PFA at 4ºC.

Wholemount stimulation, immunoprecipitation and Western Blotting

Mice were sacrificed by avertin overdose and subependymal wholemounts were dissected and either maintained for pharmacological manipulation or fixed and

stained for histology as previously described (Mirzadeh et al., 2010). Unfixed wholemounts were rapidly placed in 1xHBSS Complete and stimulated with either DMSO, or Phorbol 12-Myristate 13- Acetate (PMA; Acros Organics; 10 nmol/mL) for 4 minutes. Treated and control wholemounts were immediately homogenized and centrifuged at high speed for 20 minutes. For immunoprecipitation, 500 µg of total protein incubated with antibodies overnight at 4ºC. The following day, Dynabeads Protein A (Life Technologies) were added and cross linked for 2 hours at 4ºC. Beads were washed three times and boiled for five minutes in 2x SDS sample buffer supplemented with β-mercaptoethanol. For Western blotting, boiled protein samples were run on 10% SDS-PAGE gels and transferred to PVDF membrane for immunoblotting. Blots were blocked with 5% non-fat dry milk or 5% BSA for 1 hour at room temperature followed by overnight incubation with primary antibody at 4ºC, repeated washes and incubated in secondary antibody. Blots were then exposed to chemiluminescent substrate (BioRad) and imaged. Blots were stripped and reprobed with antibodies using standard procedures.

Histology

Brains harvested from mice were post-fixed overnight in 4% paraformaldehyde, embedded in 3% low melting agarose, and sectioned at 50 µm in the sagittal plane. Sections were blocked for 1 hour at room temperature with 10% goat serum and 1% Triton X 100 in 1x PBS followed by ON incubation with primary antibodies (diluted in1% goat serum and 0.3% Triton X 100 in 1x PBS) at 4ºC. Sections were then washed 3 times (5 minutes each) at room temperature and incubated for 1

hour with fluorescent probe conjugated secondary antibodies (Alexa secondaries, Invitrogen). Sections were washed, mounted onto glass slides, and coverslipped. Sections were then blocked and stained as described above. Antibodies used in this study included: anti-MARCKS (Blackshear lab), anti-phospho-MARCKS (Cell Signaling), anti-aPKC (Santa Cruz), anti-Clca3 (Adler lab), anti-GFP (Abcam), anti-Mucin (Covance), anti-α-tubulin (Sigma), anti-acetyl-tubulin (Invitrogen), anti-GFAP (Dako), anti-CD68 (AbD), and anti-4-Hydroxynonenal (Millipore).

Stains used in this study included Oil red O (Sigma), Concanavalin A, and Phalloidin (Life Technologies). PFA fixed sagittal brain sections or wholemount preparations were incubated for 10 minutes with freshly prepared 0.33% of Oil Red O (ORO) working solution in deionized water from a stock solution (0.5% ORO in 60% isopropanol). Stained tissues were rinsed at least 3 times with 60% isopropanol solution and mounted on glass slides with glycerol/ gelatin mounting medium (Sigma). For labeling glucose-mannose associated proteins and F-actin, Alexa Fluor-conjugated Concanavalin A and Phalloidin were added to the secondary antibody mixtures where appropriate, and sections were washed and coverslipped.

Cell counts and fluorescence intensity quantifications

Clca3 distribution within ECs was quantified by designation of the immunolabelling patterns as fibrillary (fibrous ring-like pattern) or punctate (spotty distribution). ECs with either fibrillary or punctate distribution patterns were counted in high resolution confocal Z-stack images obtained using a 60x objective on an Olympus Fluoview

FV1000 confocal microscope. 300 Clca3+ ECs were counted per animal and percentages were determined for each pattern of distribution.

Signal intensities for various immunostained markers were quantified in Image J using gray scale images from individual fluorophore channels in single slices of Z-stack confocal captures. For measurements in individual ECs, images were obtained with a 60x oil objective lens and digitally zoomed at 2x (Olympus FV1000, FV10-ASW 3.0 Viewer). Five equidistant regions of interest were drawn along the apico-basal axis of each ependyma and recordings were transferred to an Excel file for analysis. For measurements of mucin intensity in sagittal sections, 10 equidistant bins circumscribed around the lateral ventricles were delineated for measurements in tiled images obtained using a 10x and 20x objectives on the Olympus confocal using its Multi Area Time Lapse module. Background measurements on the same images from surrounding tissue with no fluorescent labeling were subtracted from readouts. Signal intensities were either presented on a normalized scale of 0 (no signal) to 2.5 (saturated signal), or as a percentage of total signal for each region of interest.

Planar Clca3 signal intensity measurements were obtained using Fluoview FV10-ASW software (version 4.1) from high resolution confocal Z-stack images of wholemount surfaces. Signal intensity values were obtained at equidistant intervals spanning the medio-lateral aspects of individual ependymal cells (average intervals, 0.1 µm) and normalized for comparisons across individual cells and wholemounts from three different mice per age and genotype. Values were obtained from lines drawn across individual ECs and plotted along the normalized

planar lengths including lateral and midline aspects of ECs. Only data from lines crossing the nucleus were included to avoid biased measurements from the lateral surfaces of ECs.

Oil Red O stained sections and wholemounts were imaged using a Zeiss fluorescent microscope. Numbers of ORO labeled droplets were determined by systematic counting in 50 µm x 50 µm grids laid over images obtained from wholemounts. Final numbers were normalized to counts per mm^2.

For all data, statistical significance relative to 2M-WT data was determined using Student's t-test.

Electron microscopy

Mice were transcardially perfused using McDowell's and Trump's 4F:1G fixative (McDowell and Trump, 1976) and dissected brains were post-fixed in 4F:1G overnight at 4ºC. The ventricular zone was microdissected from 50 µm sagittal sections using a surgical blade. Dissected tissues were ultrathin sectioned, processed, and imaged for electron microscopic analysis at the Laboratory for Advanced Electron and Light Optical Methods (LAELOM) at the College of Veterinary Medicine, North Carolina State University.

Ependymal barrier function assay

Ependymal wholemounts were harvested from mice and mounted in 0.012 cm^2 aperture tissue cassettes, mounted in Ussing chambers (Physiologic Instruments, Inc, San Diego, CA) for measurement of epithelial permeability in terms of

transepithelial electrical resistance (TER) and FITC dextran permeability as previously described (Smith et al., 2010). While on the Ussing chambers, ependymal wholemounts were bathed in oxygenated Ringer solution and maintained at 37°C via a circulating water bath. 10 mM glucose was added to the chamber in contact with the basal (interstitial) side of the wholemount, and was osmotically balanced with 10 mM mannitol on the apical (CSF) chamber (Fig. 5A). After a 15 minute stabilization period, FITC-dextran (FD4; 2.2 mg/mL, 4.4 kDa; Sigma) was added to the apical reservoir. After another 15 minutes of equilibration, 100 µL samples (in triplicates) were collected from the basal side of wholemounts at 15 minute intervals for 30 minutes and transferred into a 96 well assay plate. The presence of FD4 was assayed by measurement of fluorescence intensity using an fMax Fluorescence Microplate Reader (Molecular Devices, Sunnyvale, CA) and concentrations were determined from standard curves generated by serial dilution of FD4. FD4 flux rates were calculated by subtracting the initial FD4 concentration from final FD4 concentration at the end of 30 minute period and presented as FD4 flux rate in mg/min.

Supporting Information References

Barnabe-Heider F, Meletis K, Eriksson M, Bergmann O, Sabelstrom H, Harvey MA, Mikkers H, Frisen J (2008) Genetic manipulation of adult mouse neurogenic niches by in vivo electroporation. *Nat Methods* **5**,189-196.

El Zein L, Ait-Lounis A, Morle L, Thomas J, Chhin B, Spassky N, Reith W, Durand B (2009) RFX3 governs growth and beating efficiency of motile cilia in mouse and controls the expression of genes involved in human ciliopathies. *J Cell Sci* **122**,3180-3189.

Fang S, Crews AL, Chen W, Park J, Yin Q, Ren XR, Adler KB (2013) MARCKS and HSP70 interactions regulate mucin secretion by human airway epithelial cells in vitro. *Am J Physiol Lung Cell Mol Physiol* **304**,L511-L518.

Guirao B, Meunier A, Mortaud S, Aguilar A, Corsi JM, Strehl L, Hirota Y, Desoeuvre A, Boutin C, Han YG, Mirzadeh Z, Cremer H, Montcouquiol M, Sawamoto K, Spassky N (2010) Coupling between hydrodynamic forces and planar cell polarity orients mammalian motile cilia. *Nat Cell Biol* **12**,341-350.

Jacquet BV, Muthusamy N, Sommerville LJ, Xiao G, Liang H, Zhang Y, Holtzman MJ, Ghashghaei HT (2011) Specification of a Foxj1-Dependent Lineage in the Forebrain Is Required for Embryonic-to-Postnatal Transition of Neurogenesis in the Olfactory Bulb. *J Neurosci* **31**,9368-9382.

Jacquet BV, Patel M, Iyengar M, Liang H, Therit B, Salinas-Mondragon R, Lai C, Olsen JC, Anton ES, Ghashghaei HT (2009a) Analysis of neuronal proliferation, migration and differentiation in the postnatal brain using equine infectious anemia virus-based lentiviral vectors. *Gene Ther* **16**,1021-1033.

Jacquet BV, Salinas-Mondragon R, Liang H, Therit B, Buie JD, Dykstra M, Campbell K, Ostrowski LE, Brody SL, Ghashghaei HT (2009b) FoxJ1-dependent gene expression is required for differentiation of radial glia into ependymal cells and a subset of astrocytes in the postnatal brain. *Development* **136**,4021-4031.

McDowell EM, Trump BF (1976) Histologic fixatives suitable for diagnostic light and electron microscopy. *Arch Pathol Lab Med* **100**,405-414.

Mirzadeh Z, Doetsch F, Sawamoto K, Wichterle H, Alvarez-Buylla A (2010) The subventricular zone en-face: wholemount staining and ependymal flow. *J Vis Exp*.

Smith F, Clark JE, Overman BL, Tozel CC, Huang JH, Rivier JE, Blikslager AT, Moeser AJ (2010) Early weaning stress impairs development of mucosal barrier function in the porcine intestine. *Am J Physiol Gastrointest Liver Physiol* **298**,G352-G363.
