# Supplementary material for: MARCKS-dependent mucin clearance and lipid metabolism in ependymal cells are required for maintenance of forebrain homeostasis during aging
Source: Aging Cell. 2015 May 25;14(5):764–73. doi: 10.1111/acel.12354 (PMC4568964; doi:10.1111/acel.12354)
Supplement: Supplementary file 12 [file acel0014-0764-sd12.pdf]

Movie S1. Movie of confocal Z-stacks scanned in sagittal brain slices immunostained for MARCKS (red) of 2M-WT ECs (green) before and after PMA treatment.

Movie S2. Movie of confocal Z-stacks scanned in sagittal brain slices immunostained for p-MARCKS (red) in 2M-WT ECs (green) before and after PMA treatment.

Movie S3. Movie of confocal Z-stacks scanned in sagittal brain slices immunostained for aPKC (red) in 2M-WT ECs (green) before and after PMA treatment.

Movie S4. Movie of confocal Z-stacks scanned in sagittal brain slices immunostained for MARCKS (red) of 2Y-WT ECs (green) before and after PMA treatment.

Movie S5. Movie of confocal Z-stacks scanned in sagittal brain slices immunostained for p-MARCKS (red) in 2Y-WT ECs (green) before and after PMA treatment.

Movie S6. Movie of confocal Z-stacks scanned in sagittal brain slices immunostained for aPKC (red) in 2Y-WT ECs (green) before and after PMA treatment.

Movie S7. Time-lapse confocal imaging of Fc:tdTom+ ECs (red) cultured for 28 days after electroporation with a MARCKS::YFP construct. MARCKS::YFP

(green) robustly associates with the membrane of ECs and appears unaffected upon vehicle (DMSO) treatment. Addition of PMA to the culture medium immediately stimulates dissociation of MARCKS from the membrane and its intracellular transport to vacuole-like structures (round organelles) in ECs.

Movie S8. Time-lapse imaging of an acute 2M-WT ependymal wholemount preparation maintained ex vivo, and expressing MARCKS::YFP (gray) before and after PMA treatment.

Movie S9. Time-lapse imaging of an acute 2Y-WT ependymal wholemount preparation maintained ex vivo, and expressing MARCKS::YFP (gray) before and after PMA treatment..
